# Prevention, Treatment, and Monitoring of Seizures in the Intensive Care Unit

**DOI:** 10.3390/jcm8081177

**Published:** 2019-08-07

**Authors:** Micheal Strein, John P. Holton-Burke, LaTangela R. Smith, Gretchen M. Brophy

**Affiliations:** 1 Department of Pharmacotherapy and Outcomes Science, Virginia Commonwealth University School of Pharmacy, Richmond, VA 23298-0533, USA; 2 Department of Neurology, Virginia Commonwealth University Health System, Richmond, VA 23298-0599, USA

**Keywords:** neurocritical care, critical care, seizures, status epilepticus, electroencephalography, antiepileptic therapy

## Abstract

The diagnosis and management of seizures in the critically ill patient can sometimes present a unique challenge for practitioners due to lack of exposure and complex patient comorbidities. The reported incidence varies between 8% and 34% of critically ill patients, with many patients often showing no overt clinical signs of seizures. Outcomes in patients with unidentified seizure activity tend to be poor, and mortality significantly increases in those who have seizure activity longer than 30 min. Prompt diagnosis and provision of medical therapy are crucial in order to attain successful seizure termination and prevent poor outcomes. In this article, we review the epidemiology and pathophysiology of seizures in the critically ill, various seizure monitoring modalities, and recommended medical therapy.

## 1. Introduction

Seizures and status epilepticus (SE) have a large clinical and economic impact on the care of critically ill patients worldwide as they are often associated with complicated and lengthy hospital and intensive care unit (ICU) stays [1]. Neurocritical care (NCC) is a rapidly growing specialty that specializes in the care of critically ill patients presenting with primary neurological injuries [2]. For these patients, the involvement of expert NCC clinicians has led to significantly better patient outcomes. Some of the most notable NCC specialty areas include seizures and SE, ischemic and hemorrhagic stroke, and traumatic brain injury (TBI). Although seizures are not always the initial injury, critically ill patients may develop a secondary neurological deterioration due to ongoing intracranial pathophysiologic changes and central nervous system (CNS) insults, leading to subsequent seizures or SE. The most common secondary injuries are brain tissue hypoperfusion, brain tissue hypoxia, and excitotoxic damage due to recurrent seizures [3]. This article will focus on the epidemiology and pathophysiology of seizures in critically ill patients, as well as how monitoring and therapeutic strategies can aid in diagnosing and treating primary and secondary seizures and SE in this challenging population.

## 2. Epidemiology

The published incidence of seizures in critically ill patients is highly variable but has been reported to range from 8% to 34% based on continuous electroencephalography (EEG) monitoring studies published from 1994 to 2011 [4]. The most common comorbidities and conditions associated with seizure in critical illness include a pre-existing history of epilepsy, direct CNS insults, metabolic derangements, and drug withdrawal or intoxication [4,5] (Table 1). Of the many potential CNS insults, those most frequently associated with seizures are CNS infection, stroke, brain tumor, and neurosurgical procedures [4,5]. In critically ill patients with seizures, SE must always be considered and even anticipated. This is especially true in comatose patients and those without return to baseline or with waxing/waning mentation. The likelihood of capturing seizure on continuous EEG is highest in younger patients, those with pre-existing epilepsy, prior neurosurgical procedure, and convulsion or comatose state prior to the start of continuous EEG monitoring [5]. Of seizures captured in one study, 34% were nonconvulsive seizures (NCSz), and of these, 76% were nonconvulsive status epilepticus (NCSE) [5]. 

SE is defined as 5 or more minutes of continuous clinical and/or electrographic seizure activity, or recurrent seizure activity without recovery between seizures. Patients who do not respond to standard treatment regimens for SE (i.e., benzodiazepine and an anticonvulsant drug) are considered to be in refractory SE (RSE). Cases where SE continues for 24 h or more after the initiation of anesthetic therapy, including those where SE recurs during reduction or withdrawal of anesthesia, are considered to be in super-refractory SE (SRSE) [10,11]. The annual incidence of SE in the United States (US) and worldwide is 100,000 to 152,000 and 1.2 to 5 million, respectively [12]. Young, African American males appear to have a higher incidence of SE but lower associated mortality [6]. In a multicenter cohort by Shin et al., SE was most commonly associated with cerebrovascular disease, substance use, and CNS inflammation [7]. CNS inflammation was due to infection, autoimmune encephalitis, or cryptogenic [7]. Of these, cryptogenic CNS inflammation leading to SE was most challenging to treat and considered an independent risk factor for SRSE [7]. In the US, the most common comorbidities associated with SE are consistent with those associated with seizures in critically ill patients, with the inclusion of cerebral anoxia and congenital disorders [6]. If the workup for these conditions is negative, there should be high suspicion for cryptogenic new-onset refractory status epilepticus (NORSE) and autoimmune/paraneoplastic syndromes. Liu et al. found that in patients with anti-*N*-methyl-d-aspartate receptor (NMDAR) encephalitis, 80.7% had seizure during the acute phase of the disease [13]. Fifty percent of those with seizure developed SE, with 25% of these being refractory to initial treatment requiring multiple anticonvulsants plus anesthetic agents (midazolam/propofol) [13]. Over one-third of the refractory cases were termed SRSE due to inability to withdraw or reduce anesthetic agents and resulted in patient death [13].

Neurological injuries or secondary neurological injuries from other disease states can also lead to SE (Table 1). In cardiac arrest patients, the major cause of death is hypoxic ischemic brain injury sustained during the arrest [14]. However, a high proportion of the patients that obtain return of spontaneous circulation go on to develop seizures or SE post-resuscitation [15]. It is not known if SE contributes to poor outcomes after cardiac arrest or if it is a consequence of the severe brain injury, and overall EEG monitoring is currently of unclear benefit in regard to patient outcomes [16]. 

## 3. Pathophysiology 

Seizure results from abnormally excessive, neuronal activity as a consequence of the disrupted balance between neuronal excitation and inhibition [17]. What leads to disruption of this balance is not always known. Fairly recognized culprits involve breakdown of the blood–brain barrier (BBB) and profound metabolic or electrolyte imbalance (i.e., hypoglycemia, hyponatremia/hypernatremia). Given that CNS infection, brain tumor, and cerebral hypoxia/ischemia are among the most common comorbidities associated with seizures in critically ill patients, it can be postulated that cerebral inflammation is at least a secondary if not direct cause of disrupted neuronal activity. Further, it has been found that cerebral inflammation is directly epileptogenic, serving to precipitate and prolong seizures [18]. There is also the question of whether peripheral inflammation in and of itself can lower seizure threshold. Experimental studies have revealed the ability of the CNS to mirror the peripheral immune response to inflammation and trigger a pro-inflammatory signaling cascade resulting in increased epileptogenicity [19,20]. Given that critical illness coincides with an increased inflammatory state, these individuals are at increased risk of seizure for the sole fact that they are acutely ill. 

Tumor necrosis factor alpha (TNF-α) is a pro-inflammatory cytokine released by activated microglia in response to infection, neurological disease, or tissue damage, and has been found to increase the permeability of the BBB when studied during in vivo experimentation [21,22]. In a study comparing serum levels of TNF-α and interleukin 4 (IL-4) in patients with febrile seizures versus controls (febrile patients without seizure or history of febrile seizures), it was found that both TNF-α and IL-4 concentrations were significantly higher in patients with febrile seizures [23]. The fact that both cytokines were elevated in the febrile seizure patients seems somewhat contradictory, as IL-4 is anti-inflammatory [24]. However, it is hypothesized that upregulation of defense cytokines, such as IL-4, as concentrations of pro-inflammatory cytokines rise, is a part of the pathogenicity of seizure [23]. Additionally, it is difficult to refute the crucial role inflammation plays in epileptogenesis given that Rasmussen encephalitis involves persistent cerebral inflammation and intractable seizures [25]. In a pilot study evaluating the effectiveness of adalimumab (anti-TNF-α antibody) in treating Rasmussen encephalitis, a reduction in seizure frequency was noted in patients taking the drug [26]. An experimental study by Riazi et al. was also able to demonstrate the anti-epileptic effect of anti-inflammatory therapy during inflammatory disease [19]. In this study, it was found that rats with induced colonic inflammation were more likely to seize and were also found to have hippocampal tissue with a significantly higher amount of activated microglial cells than controls [19]. Additionally, administration of minocycline resulted in a decrease in this hippocampal excitability despite having no significant effect on colonic inflammation [19]. Ultimately, whether peripheral or central, inflammation can alter CNS excitability leading to reduced seizure thresholds.

When evaluating for causes of seizure in critically ill patients, it remains crucial to thoroughly review common reversible causes, such as medication withdrawal, medication-induced seizures, substance abuse, and metabolic derangements. Metabolic derangements (hypoglycemia, uremia, alterations in serum osmolality, electrolyte imbalance) are frequently encountered in the critical care setting [27]. Electrolyte balance is critical because it directly affects the ionic gradient across neuronal cells, which, in turn, directly affects cellular excitability. The electrolyte abnormalities most frequently resulting in seizures include hyponatremia, hypernatremia, hypocalcemia, and hypomagnesemia [28]. Performing due diligence in obtaining an accurate home medication list is critical to ensure that medications that can potentiate seizure if withheld are continued if no contraindications exist. Though abrupt withdrawal of anticonvulsants, benzodiazepines, and barbiturates is an obvious cause of seizure, withdrawal of other medications such as baclofen, opiate analgesics, and sedative/hypnotics (e.g., zolpidem) has also been implicated in increasing seizure susceptibility [17]. Introduction of new medications should also be considered as a possible culprit, as there are many medications believed to lower seizure threshold (Table 2). 

Due to the varied causes of seizure in critically ill patients, thorough investigation should be implemented in each case. This involves correction of any metabolic disturbances, extensive medication and medication history review, and toxicology screening. If no immediate reversal cause is identified, further neuroimaging and likely CSF studies would be warranted. Lastly, aggressive treatment of acute illness should not be discounted given that an acute inflammatory state in and of itself can lower seizure threshold.

## 4. Monitoring 

Many different seizure monitoring devices have been increasingly utilized in the critical care setting; however, there is a paucity of literature providing guidance on when to use which modality. Continuous EEG monitoring has shown a significant increase in utilization in the NCC setting [32]. This is especially important in patients with unexplained altered mental status and histories of epileptic and non-epileptic seizures, as a significant amount of nonconvulsive seizures have been detected in critically ill patients [5,33,34]. Recent investigations have shown that a traditional scalp EEG recording may not be sufficient to appropriately diagnose seizures in the critically ill and may be missing other causes of neurological deterioration in this population of patients [35]. Many clinicians have increased monitoring to aid the understanding of brain physiology in real time. This allows for early detection of physiologic and electrochemical disturbances that can be promptly treated to salvage viable tissue at risk of secondary injury. This is clinically important as NCSz and NCSE are associated with higher morbidity and mortality [36].

Seizure monitoring modalities have been rapidly improving and are now employing both extracranial and intracranial systems. The benefits of using an extracranial system are lack of invasive procedures, ease of application, and prompt monitoring. The move from routine EEGs to continuous EEGs was noted as conventional EEGs found seizures in 11% of critically ill patients, while prolonged monitoring found seizures in 27% [37]. This was most prevalent in the first 24 h of admission, but longer recordings may be required in comatose patients, or those with abnormalities noted on EEG. Recently, there has been a significant increase in the utilization of continuous EEG and could be viewed as a requirement in newly admitted critically ill patients with altered mental status as well as epileptic seizures [4]. Prolonged EEG recordings will initially be targeted to show slowing, cortical depression, periodic discharges, or epileptic seizures. This is often a qualitative target which requires the expertise of an experienced reader of EEGs and is a useful adjunct in patients who are not improving as expected clinically [35]. 

Additional information obtained via quantitative EEG (qEEG) methodology using scalp electrodes may also be beneficial. Real-time qEEG is a computerized analysis of the digitized EEG, which allows a modified brain mapping to be interpreted by the electroencephalographer [38]. The qEEG can provide valuable information regarding focal slowing, frontal lobe disturbances, low magnitudes, interictal activity, as well as brain asymmetry [39]. Along with the qEEG system, the emergence of off-site interpretation of EEGs via a cloud based system, or tele-EEG (tEEG), has been shown to be a feasible, secure, and timely method of providing EEG service to hospitals which cannot always staff 24/7 coverage [40]. Moreover, training ICU nursing staff and clinical pharmacists to recognize the alarm system could allow a more rapid analysis of the qEEG data associated with potential seizure activity and treatment escalation, as appropriate. Other alternatives to qEEG for providers who are not trained to interpret brain wave activity and/or for possible NCSE have also recently come to the market and have shown clinical efficacy, ease of use, and rapid acquisition [41]. 

Unfortunately, recent data have shown that a continuous EEG alone may not be sufficient to detect deep foci of seizures or other unexplained deteriorations, at which point a high level of monitoring may be indicated. Intracortical monitoring has shown that in patients with unexplained neurological declines, up to 60% of seizing patients may not have scalp EEG correlates [42]. This leads to the need for either high-density EEG (HDEEG) or intracranial monitoring as treatment escalates but is typically driven by the available resources at the treating facility. HDEEG does show the benefit of increasing epileptic spike detection by as many as threefold, with up to 90% of temporal lobe spikes not being found using traditional 10–20 EEG montages [43,44]. 

The gold standard of spike and seizure detection remains intracranial monitoring; however, weighing the benefits and risks of this modality reserves this for the most critically ill patients [44]. Intracranial monitoring is now being employed using either craniectomy and grid placement or a cranial bolt system. With a triple or quadruple bolt system, many different parameters can be measured simultaneously [45]. In addition to intracranial EEG, monitoring other variables can be added, including intracranial pressure, cerebral blood flow, microdialysis, and brain oxygen probes which can detect brain tissue hypoxia, intracerebral metabolic derangements, and more quantitative information on the brain tissue being monitored [3]. Complications to probe placement are less than 11% according to some sources and are generally procedure-related hemorrhage, infection, or misplacement of the probe [46]. Inevitably, invasive monitoring is associated with higher complication rates as compared to scalp electrodes and HDEEG [46]. Intracranial monitoring should be reserved for patients who have unexplained alterations of mental status after undergoing continuous video EEG and risk factor modification. Due to the risks of invasive monitoring, if seizures are still suspected in spite of an unrevealing EEG, it is reasonable to trial a short course of benzodiazepines, such as lorazepam, while observing for improvement in mental status. Long-term antiseizure therapy has not been shown to improve a patient’s hospital course without a clinical indication; in fact, quite the opposite. Thus, therapy should not be continued indefinitely.

## 5. Treatment

The provision of an anticonvulsive agent for seizure prophylaxis in various disease states is something that is still widely debated. There is a paucity of data to guide clinicians as to which disease states should receive prophylaxis, which agent to use, and the optimal duration of prophylaxis. As discussed previously, the incidence of seizures in critically ill patients is highly variable; however, seizure prophylaxis is typically utilized in TBI, aneurysmal subarachnoid hemorrhage (aSAH), intracerebral hemorrhage, brain neoplasm, and postoperatively after craniotomy [47,48,49]. Current guidelines only support the routine use of seizure prophylaxis in patients with severe TBI and suggest consideration for use following aSAH [50,51]. Despite the indication, seizure prophylaxis should only be used to prevent early seizures (within 7 days), as data have not shown a benefit of prophylaxis in late-onset seizures (>7 days after incident) [52,53,54]. The Brain Trauma Foundation guidelines specifically cite use of phenytoin for prophylaxis after severe TBI due to lack of data with other agents [51]. The American Heart Association/American Stroke Association guidelines on aSAH do not cite a specific agent, although the Neurocritical Care Society recommends against the routine use of phenytoin for this indication, citing possible worse outcomes [50,51,55]. If a patient has a confirmed seizure at any point during hospitalization, treatment should then be instituted and continued as long as clinically indicated, as the patient is at a greater risk of recurrent seizures.

**Figure 1:**
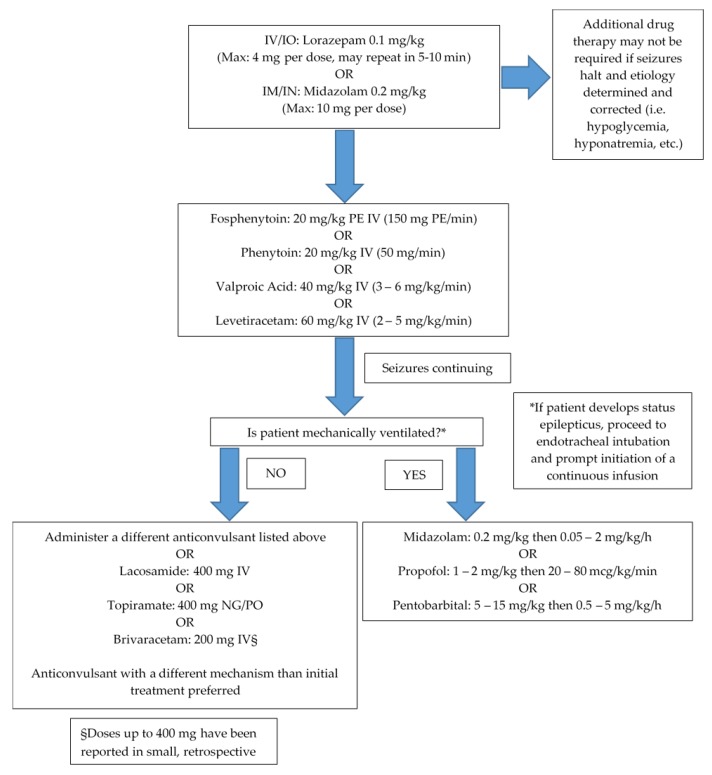
Seizure and status epilepticus treatment algorithm for critically ill patients [10,56,57,62,63,64,65,66,67,68].

Management of seizures in the critically ill typically follows a stepwise approach (Figure 1). Initial treatment should consist of prompt administration of adequately dosed benzodiazepines. Lorazepam and midazolam are the preferred agents for intravenous (IV) and intramuscular (IM) administration, respectively [10,56,57]. Diazepam, while historically used, is not preferred for initial therapy if lorazepam or midazolam are readily available. Diazepam has a large volume of distribution which results in rapid redistribution of drug out of the central nervous system to adipose tissue [6]. This redistribution may result in subtherapeutic concentrations and seizure recurrence if additional anticonvulsants are not promptly administered (e.g., within 30 min). If IV access has not been obtained or has been lost during convulsive activity, midazolam may be administered IM or intranasally (IN). Intranasal administration should be performed with the use of a mucosal atomization device using the same dosing strategy as IM and IV dosing. The midazolam 5 mg/mL IV product is recommended for this route to minimize volume, and the total dose administered should be equally divided between each nostril [58,59]. Intraosseous (IO) administration of midazolam or lorazepam may also be considered if other routes of administration are not feasible. Standard practices for IO insertion should still be applied, and placement should be verified by aspiration of a small amount of bone marrow followed by administration of 5–10 mL of 0.9% sodium chloride to ensure lack of resistance and to clear the needle [60]. Regardless of the medication used and route of administration, timing of medication administration and appropriate dosing are of utmost importance. As seizure activity continues, synaptic gamma-aminobutyric acid (GABA) receptors (benzodiazepine pharmacologic target) begin to internalize, resulting in a decreased efficacy of benzodiazepine therapy [61]. Some clinical concerns exist over the large doses of benzodiazepines recommended for termination of seizure activity with regard to respiratory compromise. However, studies in the prehospital setting have shown that the need for placement of an advanced airway is more likely related to continued seizure activity rather than the benzodiazepines administered at the recommended doses [56,57].

Following administration of benzodiazepine, patients should be treated with longer acting anticonvulsants to aid in seizure cessation in those still seizing despite appropriately dosed benzodiazepine therapy, or to prevent recurrent seizures in those who have achieved successful seizure termination [10]. Patients who have had a treatable cause of seizure identified and corrected do not require additional therapy with an anticonvulsant (i.e., hypoglycemia, hyponatremia, etc.). There is no consensus on which anticonvulsant to administer after benzodiazepine therapy, so the decision should be patient-specific. Factors to consider include potential adverse drug effects, drug–drug interactions, hemodynamic stability, renal and/or hepatic dysfunction, serum albumin, previous history of anticonvulsant use, and therapeutic monitoring availability both in the inpatient and outpatient setting. Important properties of anticonvulsant medications are noted in Table 3. All agents should initially be administered parenterally as loading doses to rapidly attain therapeutic serum concentrations. Considerations for initiating oral maintenance therapy include what agent terminated seizures, enteral formulation compatibility (as applicable), and concern for decreased drug absorption (e.g., high-dose vasopressor therapy, septic shock). In general, the older anticonvulsants have more data supporting clinical efficacy and can be monitored using serum drug concentrations, but at the cost of more adverse drug reactions and drug–drug interactions. The newer anticonvulsants have less clinical data supporting their efficacy, especially in SE, and ill-defined therapeutic serum concentrations, but tend to have fewer adverse effects and drug–drug interactions. Thus, there are many controversies over which anticonvulsant agent is the best for treating seizures in critically ill patients. 

Patients who have continuous seizure activity for 5 min, or at least 2 seizures without return to baseline between seizures, are considered to be in SE, as discussed previously. Due to the significant mortality and morbidity associated with this medical emergency, prompt and aggressive treatment is recommended [10,72]. To ensure timely administration, benzodiazepines and premade, ready-to-use anticonvulsant products should be available in automated dispensing cabinets in ICUs as well as the emergency department, unless a satellite pharmacy is located in close proximity. Additionally, prebuilt order sets, following the Institute for Safe Medication Practices (ISMP) recommendations, should be implemented for ease of appropriate medication ordering [73]. Patients whose seizures continue after administering appropriately dosed benzodiazepines and anticonvulsants are now considered to be in RSE. Establishing an institution-specific protocol detailing which anesthetic medications to utilize for RSE is also advised, as these agents are typically dosed higher than in other disease states. 

## 6. Special Considerations

The use of continuous renal replacement therapy (CRRT) in critically ill patients is an intervention that is becoming more and more common. Unfortunately, there is a relative paucity of data available evaluating anticonvulsant dosing in patients on any modality of CRRT (Table 4). For this reason, any anticonvulsants that have readily available serum monitoring assays should be utilized in this setting to assist in guiding medication dosing [69,74]. If a serum drug assay is not available, there are a few factors that should be considered. Generally, a drug that is eliminated renally will be removed using CRRT. The degree of removal will largely depend on the CRRT flow rate and modality (e.g., continuous venovenous hemofiltration and/or hemodialysis), the degree of protein binding (only unbound drug will be removed by CRRT), and the volume of distribution of the drug [74]. Other important considerations for anticonvulsant dosing in patients on CRRT is monitoring for any filter down time as well as flow rate changes, as these may warrant further modification to drug dosing. While molecular weight of the drug is often cited as a consideration, the weights of available anticonvulsants are all small enough that this factor is not of clinical significance. Unfortunately, there is currently not enough evidence to recommend specific anticonvulsant doses in those undergoing CRRT; therefore, the clinician must consider the characteristics of each drug when making dosing decisions, and serum drug levels should always be utilized, if available [69,74,75]. Additionally, the development of any adverse effects believed to be related to an anticonvulsant should prompt dosing modifications. 

Similar to drug dosing in CRRT, data regarding drug dosing in patients with acute liver failure are also lacking (Table 4). Serum drug concentrations should be followed closely when available in this unique patient population. Dosing recommendations based on Child–Pugh scores may also be considered, keeping in mind that these dosing recommendations were designed for patients with chronic liver disease [76]. If an anticonvulsant is to be initiated in the setting of acute liver failure, it is best to avoid those with low hepatic extraction ratios (i.e., phenytoin, valproic acid, and phenobarbital) as clearance of these drugs will be primarily predicated upon intrinsic hepatic function. 

The use of extracorporeal membrane oxygenation (ECMO) continues to rise in critically ill patients, but unfortunately, the literature surrounding specific drug dosing remains sparse. The biggest impact of ECMO on drug dosing lies in the propensity for the ECMO circuit to sequester drugs, resulting in a larger than expected volume of distribution. This phenomenon may decrease over time with continued dosing due to saturation of binding sites. In general, medications with a higher degree of lipophilicity and protein binding tend to be sequestered more [77,78]. Many patients on ECMO frequently receive concomitant CRRT, which further complicates the dosing picture and heightens the need for therapeutic drug monitoring, if available. 

Critically ill patients regularly receive numerous medications during their stay in the ICU. In the patient receiving an anticonvulsant drug, medication lists should be carefully scrutinized for drug–drug interactions, as many of these medications are inhibitors or inducers of certain metabolic enzymes. Fosphenytoin/phenytoin, phenobarbital, and pentobarbital are all enzyme inducers and may reduce the concentration of concurrent medications [79]. Additionally, anticonvulsant drugs with high degrees of protein binding may displace other medications with a high degree of protein binding, resulting in an increased free fraction of one or both medications which may precipitate adverse effects. The potential for this is heightened in critically ill patients who often have reduced plasma albumin and acid/base abnormalities [80,81,82]. One additional serious interaction to consider as the incidence of multidrug-resistant organisms increases is between carbapenem antibiotics and valproic acid. Numerous potential mechanisms exist to describe this interaction; however, it is believed that carbapanems inhibit an enzyme crucial to the production of the pharmacologically active moiety of valproic acid, resulting in significantly reduced plasma valproic acid concentrations [83,84,85]. In patients on valproic acid, alternatives to carbapenems should be utilized if possible. If a carbapenem must be used, patients should be started on another anticonvulsant drug prior to initiation. 

## 7. Older Adults, Pediatrics, and Pregnancy

The initial management approach of acute onset seizures in the critically ill older adult and pediatric populations is similar to that of other adult patients. Medication dosing is primarily weight-based, and there are generally no modifications required for the initial dosing strategy. However, a number of physiological changes occur in older adults that may affect the pharmacokinetics of anticonvulsants, including possible decrease in drug absorption, increase in total body fat, decrease in total body water, and reduced hepatic and renal function [86]. Older adult patients may also have increased blood–brain-barrier permeability, which lends to a higher risk of adverse effects associated with anticonvulsant use [87]. When considering anticonvulsants in this population, it is important to consider each of these aspects, as well as concomitant disease states and medications. 

Many of the older anticonvulsants that undergo hepatic metabolism may be enzyme inhibitors or inducers. These may affect or be affected by other medications, altering serum concentrations and potentially leading to sub- or supratherapeutic concentrations. Therefore, in the older adult patient on numerous medications with potential for interactions, it may be prudent to assess serum drug concentrations more frequently than in other patients to ensure efficacy and safety. In patients receiving newer anticonvulsants for which target serum concentrations are not as well defined, dose modifications should be made based on estimated renal function using the Cockcroft–Gault equation and corresponding doses listed in the package inserts. Identifying anticonvulsant agents with a lower incidence of dizziness and ataxia, especially when transitioning to home care, is also important due to the higher risk of falling in the older adult patient population. 

Many pharmacokinetic properties are different in the pediatric population and change as the patient ages. Neonates tend to have relatively reduced fat compared to adults, whereas infants tend to have increased fat. This results in an increased volume of distribution for lipophilic drugs in infants and a decreased volume of distribution for lipophilic drugs in neonates. Pediatric patients also have reduced plasma proteins, leading to a lower degree of protein binding and a higher degree of free drug in anticonvulsants with high protein binding. Metabolism and elimination are also effected due to larger relative liver and kidney sizes, resulting in a greater degree of metabolism of drugs that are extensively hepatically metabolized and increased clearance of renally eliminated drugs, respectively [88]. Some medications have increased risk of toxicity in children (e.g., valproic acid–hepatotoxicity); therefore, the risk versus benefit of each treatment strategy should be considered.

Critically ill pregnant patients who experience seizures or SE should be treated aggressively to halt seizure activity so additional complications are limited. Agent selection is important over the long term due to potential teratogenic effects, but acute and chronic pharmacokinetic alterations also need to be considered. During pregnancy, the plasma volume is expanded by approximately 50%, resulting in an increased volume of distribution. As pregnancy progresses, this volume expansion leads to a relative dilutional hypoalbuminemia and may result in a greater free fraction of drugs that are highly bound to albumin (e.g., phenytoin, valproic acid). The hepatic metabolism of phenytoin has been reported to increase and is possibly due to increased microsomal enzyme activity induced by progesterone, so this should be considered for other hepatically metabolized anticonvulsants as well. As a result of the increased cardiac output during pregnancy, renally eliminated anticonvulsants have an increased clearance [89]. 

Aside from the changes in pharmacokinetics during pregnancy, the potential for a medication to cause teratogenicity should also be evaluated. In the acute setting, it is best to avoid initiation of valproic acid as it has the most data suggesting it is teratogenic. Other anticonvulsants with high teratogenic potential include phenytoin, phenobarbital, and topiramate. Lamotrigine and levetiracetam are the most commonly recommended anticonvulsant agents for pregnant patients based on the amount of evidence showing lower teratogenic risk. 

## 8. Summary

The occurrence of seizures and SE in the critically ill may be attributed to a variety of factors, while the true incidence remains unknown. Increased use of prolonged monitoring techniques as well as the development of more advanced monitoring systems may aid in bridging this knowledge gap. Further comprehension of seizure and SE incidence in critically ill patients may also allow for improved delineation of the need for seizure prophylaxis, as this concept still remains heavily debated. For optimal outcomes, early recognition of seizure activity followed by prompt, appropriately dosed medication therapy remains the hallmark of treating acute onset seizures and SE in critically ill patients. 

## Figures and Tables

**Table 1:** Neurological conditions associated with seizures and status epilepticus in critically Ill patients [4,5,6,7,8,9].

Condition	
Pre-existing epilepsy	Traumatic brain injury
Central nervous system infection	Ischemic stroke
Brain tumor	Hypoxic ischemic encephalopathy
Neurosurgical procedure	Altered mental status
Intracerebral hemorrhage	Drug toxicity/withdrawal
Subarachnoid hemorrhage	Toxic metabolic encephalopathy
Subdural hemorrhage	Congenital

**Table 2:** Common medications that may lower seizure threshold [29,30,31].

Medication Class	Select Medications
Antimicrobials	Carbapenems (imipenem, meropenem), cephalosporins (cefepime), fluoroquinolones (levofloxacin), macrolides (erythromycin), penicillins, isoniazid, linezolid, metronidazole, amphotericin, fluconazole, mefloquine, chloroquine, pyrimethamine, acyclovir, ganciclovir, foscarnet
Analgesics	Alfentanyl, codeine, fentanyl, meperidine, morphine, NSAIDs, pentazocine, tramadol
Antihistamines	Cyproheptadine, promethazine
Antiasthmatics	Albuterol, aminophylline, theophylline, terbutaline
Antineoplastics	Alkylating agents (busulfan, carmustine, chlorambucil), Platinum analogs (cisplatin), cytarabine, methotrexate, vinblastine, vincristine
Anesthetics	Bupivacaine, etomidate, lidocaine, mepivacaine, methohexital, procaine, tetracaine
Antipsychotics	Clozapine, haloperidol, lithium, olanzapine, risperidone, phenothiazines, pimozide, thiothixene
Antidepressants	Bupropion, TCAs, SSRIs, MAOIs, doxepin, trazodone, venlafaxine
Antiarrhythmics	Digoxin, flecainide
Alpha/beta agonists/antagonists	Ephedrine, esmolol, propranolol
Immunosuppressants	Cyclosporine, hydrocortisone, INF-α, methylprednisolone, Muromonab-CD3, sulfasalazine, tacrolimus
Stimulants	Dextroamphetamine, methylphenidate
Other	Atropine, baclofen, bromocriptine, desmopressin, flumazenil, levodopa, metrizamide, cyclosporine, oxytocin, sumatriptan

NSAID—Nonsteroidal anti-inflammatory drug; TCA—tricyclic antidepressants; SSRIs—serotonin reuptake inhibitors; MAOIs—monoamine oxidase inhibitors; INF-α—interferon alpha.

**Table 3:** Anticonvulsant medications [10,12,56,57,62,66,68,69,70,71].

Anticonvulsant Drug and Mechanism	Initial Dosing *	Protein Binding	Half-Life	Metabolism	Elimination	Adverse Effects
Brivaracetam SV2A modulation	100–200 mg over at least 2 min	≤20%	~9 h	Hydrolysis and hepatic via CYP2C19	>95% renally, <10% as unchanged drug	Psychiatric disturbances, nystagmus
Diazepam GABA potentiation	0.15 mg/kg (Max: 10 mg) undiluted up to 5 mg/min	98%	Parent drug: 60–72 h Metabolite: 152–174 h	Hepatic via CYP3A4 and 2C19; active metabolites	Renally as glucuronide conjugates	Respiratory depression, hypotension (more common with rapid administration)
Fosphenytoin/ Phenytoin Na^+^ channel blockade	20 mg/kg PE at 150 mg/kg/min PE 20 mg/kg at 50 mg/min	90%–95%	7–42 h	Fos: Prodrug, rapidly hydrolyzed to phenytoin. Hepatic via CYP2C9, 2C19, 3A4	<5% renally as phenytoin metabolites	Hypotension, phlebitis, cardiac arrhythmias. Consider slower administration in elderly
Lacosamide Enhances slow inactivation of voltage-gated Na^+^ channels	200–400 mg over 15–30 min	<15%	13 h	Hepatic via CYP3A4, 2C9, and 2C19; inactive metabolite	~40% renally as unchanged drug	PR interval prolongation, hypotension
Levetiracetam SV2A modulation, AMPA inhibition	3000 mg or 60 mg/kg (Max: 4500 mg) at 2–5 mg/kg/min	<10%	6–8 h	Nonhepatic hydrolysis	~66% renally as unchanged drug	Agitation, irritability, psychotic symptoms
Lorazepam GABA potentiation	0.1 mg/kg (Max: 4 mg per dose, may repeat once) up to 2 mg/min	~91%	12–18 h	Hepatic; rapidly conjugated to inactive metabolite	~88% renally as inactive metabolites	Respiratory depression, hypotension (more common with rapid administration)
Midazolam GABA potentiation	0.2 mg/kg IM (Max: 10 mg)	~97%	3 h	Extensively hepatic CYP3A4; 60% to 70% to active metabolite	~90% renally as metabolites	Respiratory depression, hypotension
Pentobarbital GABA potentiation, AMPA inhibition	5–15 mg/kg up to 50 mg/min; followed by a continuous infusion 1–5 mg/kg/h	45%–70%	15–50 h	Hepatic via hydroxylation and glucuronidation	<1% renally as unchanged drug	Respiratory depression (patient must be intubated), hypotension, constipation
Phenobarbital GABA potentiation, AMPA inhibition	15–20 mg/kg at 50–100 mg/min	50%–60%	53–118 h	Hepatic via CYP2C9 and to a lesser extent 2C19 and 2E1, and by N-glucosidation	25–50% renally as unchanged drug	Respiratory depression, hypotension, contains propylene glycol
Propofol GABA potentiation, NMDAR blockade	1–2 mg/kg followed by infusion 20–80 mcg/kg/min	97%–99%	40 min; prolonged with extended infusions	Hepatic to water-soluble sulfate and glucuronide conjugates	~90% renally as metabolites	Respiratory depression (patient must be intubated), hypotension, PRIS
Topiramate Blocks neuronal voltage-dependent Na^+^ channels, enhances GABA_A_ activity, antagonizes AMPA/kainate receptors, weakly inhibits carbonic anhydrase	200–400 mg NG/PO (not available IV)	15%–41%	19–23 h	~20% hepatically via hydroxylation, hydrolysis, and glucuronidation.	~70% renally as unchanged drug	Memory impairment, ↓ serum bicarbonate
Valproic Acid GABA potentiation, glutamate (NMDAR) inhibition, Na^+^ and Ca^2+^ channel blockade	20–40 mg/kg at 3–6 mg/kg/min	80%–90%	9–19 h	Hepatic via glucuronide conjugation and mitochondrial beta-oxidation	50–80% renally	Hepatotoxicity, pancreatitis, thrombocytopenia, hyperammonemia

* Listed as IV dosing unless otherwise stated. PE—phenytoin equivalents; AMPA—α-amino-3-hydroxy-5-methyl-4-isoxazolepropionic acid; PRIS—propofol-related infusion syndrome.

**Table 4:** Anticonvulsant dosing considerations in renal/hepatic impairment [12,69,70,71].

Anticonvulsant Drug	Renal Impairment	Hepatic Impairment
Brivaracetam	Mild to severe impairment: No dosage adjustment ESRD with HD: Not recommended (not studied)	Mild to severe impairment (Child Pugh classes A, B, and C): Initial: 25 mg twice daily, up to a max of 75 mg twice daily
Fosphenytoin/Phenytoin	No empiric dosage adjustment necessary Total serum concentration is difficult to interpret in renal failure; free concentration highly preferred	May require dosing ↓. Close monitoring of serum drug concentrations recommended
Lacosamide	CrCl ≥ 30 mL/min: No dosage adjustment necessary. Consider dose ↓ in patients taking concomitant strong CYP3A4 or CYP2C9 inhibitors CrCl < 30 mL/min: ↓ to 75% of the max dose. Further dose ↓ may be necessary with concomitant use of strong CYP3A4 or CYP2C9 inhibitors ESRD requiring HD: ↓ to 75% of the max dose. Further dose ↓ may be necessary with concomitant use of strong CYP3A4 or CYP2C9 inhibitors. Post-HD, consider supplemental dose of up to 50%	Mild to moderate hepatic impairment: ↓ dose to 75% of max dose. Further dose ↓ may be necessary in patients taking concomitant strong CYP3A4 and/or CYP2C9 inhibitors Severe hepatic impairment: Use not recommended
Levetiracetam	CrCl > 80 mL/min/1.73 m^2^: 500–1500 mg every 12 h CrCl 50–80 mL/min/1.73 m^2^: 500–1000 mg every 12 h CrCl 30–50 mL/min/1.73 m^2^: 250–750 mg every 12 h CrCl < 30 mL/min/1.73 m^2^: 250–500 mg every 12 h ESRD with HD: 500–1000 mg every 24 h; supplemental dose of 250–500 mg post-HD	No dosage adjustment necessary
Pentobarbital/ Phenobarbital	Dose ↓ recommended due to propylene glycol and potential for neurotoxicity (no specific guidance)	Dose ↓ recommended (no specific guidance)
Propofol	No dosage adjustment necessary	No dosage adjustment necessary
Topiramate	CrCl < 70 mL/min/1.73 m^2^: ↓ to 50% of normal dose and titrate slowly HD: 50–100 mg every 12 h; supplemental dose (50 to 100 mg) post-HD	No dosage adjustment necessary
Valproic Acid	No dosage adjustment necessary	Avoid

ESRD = End-stage renal Disease; HD = Hemodialysis; CrCl = Creatinine clearance.

## References

[1] Cost of Status Epilepticus: A Systematic Review—ScienceDirect. https://www.sciencedirect.com/science/article/pii/S1059131114003021.

[2] McNett M., Moran C., Johnson H. (2018). Evidence-Based Review of Clinical Trials in Neurocritical Care. AACN Adv. Crit. Care.

[3] Ko S.-B. (2013). Multimodality Monitoring in the Neurointensive Care Unit: A Special Perspective for Patients with Stroke. J. Stroke.

[4] Westover M.B., Shafi M.M., Bianchi M.T., Moura L.M.V.R., O’Rourke D., Rosenthal E.S., Chu C.J., Donovan S., Hoch D.B., Kilbride R.D. (2015). The probability of seizures during EEG monitoring in critically ill adults. Clin. Neurophysiol..

[5] Claassen J., Mayer S.A., Kowalski R.G., Emerson R.G., Hirsch L.J. (2004). Detection of electrographic seizures with continuous EEG monitoring in critically ill patients. Neurology.

[6] Brophy G.M., Bell R., Claassen J., Alldredge B., Bleck T.P., Glauser T., LaRoche S.M., Riviello J.J., Shutter L., Sperling M.R. (2012). Guidelines for the Evaluation and Management of Status Epilepticus. Neurocrit. Care.

[7] Shorvon S., Ferlisi M. (2011). The treatment of super-refractory status epilepticus: A critical review of available therapies and a clinical treatment protocol. Brain J. Neurol..

[8] Chapter 41. Status Epilepticus | Pharmacotherapy: A Pathophysiologic Approach, 9e | AccessPharmacy | McGraw-Hill Medical. https://accesspharmacy.mhmedical.com/content.aspx?bookid=689&sectionid=45310491#57485199.

[9] Dham B.S., Hunter K., Rincon F. (2014). The Epidemiology of Status Epilepticus in the United States. Neurocrit. Care.

[10] Shin J.-W., Koo Y.S., Kim Y.-S., Kim D.W., Kim K.K., Lee S.-Y., Kim H.K., Moon H.-J., Lim J.-A., Byun J.-I. (2018). Clinical characterization of unknown/cryptogenic status epilepticus suspected as encephalitis: A multicenter cohort study. J. Neuroimmunol..

[11] Liu X., Yan B., Wang R., Li C., Chen C., Zhou D., Hong Z. (2017). Seizure outcomes in patients with anti-NMDAR encephalitis: A follow-up study. Epilepsia.

[12] McNally B., Robb R., Mehta M., Vellano K., Valderrama A.L., Yoon P.W., Sasson C., Crouch A., Perez A.B., Merritt R. (2011). Out-of-hospital cardiac arrest surveillance—Cardiac Arrest Registry to Enhance Survival (CARES), United States, October 1, 2005–December 31, 2010. Morb. Mortal. Wkly. Rep. Surveill. Summ..

[13] Nielsen N., Sunde K., Hovdenes J., Riker R.R., Rubertsson S., Stammet P., Nilsson F., Friberg H., Hypothermia Network (2011). Adverse events and their relation to mortality in out-of-hospital cardiac arrest patients treated with therapeutic hypothermia. Crit. Care Med..

[14] Crepeau A.Z., Rabinstein A.A., Fugate J.E., Mandrekar J., Wijdicks E.F., White R.D., Britton J.W. (2013). Continuous EEG in therapeutic hypothermia after cardiac arrest: Prognostic and clinical value. Neurology.

[15] Won S.-Y., Dubinski D., Brawanski N., Strzelczyk A., Seifert V., Freiman T.M., Konczalla J. (2017). Significant increase in acute subdural hematoma in octo- and nonagenarians: Surgical treatment, functional outcome, and predictors in this patient cohort. Neurosurg. Focus.

[16] Pruitt P., Naidech A., Ornam J.V., Borczuk P. (2019). Seizure frequency in patients with isolated subdural hematoma and preserved consciousness. Brain Inj..

[17] Delanty N., Vaughan C.J., French J.A. (1998). Medical causes of seizures. The Lancet.

[18] Vezzani A., Balosso S., Ravizza T., Stefan H., Theodore W.H. (2012). Chapter 10—Inflammation and epilepsy. Handbook of Clinical Neurology—Epilepsy.

[19] Riazi K., Galic M.A., Kuzmiski J.B., Ho W., Sharkey K.A., Pittman Q.J. (2008). Microglial activation and TNFα production mediate altered CNS excitability following peripheral inflammation. Proc. Natl. Acad. Sci..

[20] Riazi K., Galic M.A., Pittman Q.J. (2010). Contributions of peripheral inflammation to seizure susceptibility: Cytokines and brain excitability. Epilepsy Res..

[21] Mayhan W.G. (2002). Cellular mechanisms by which tumor necrosis factor-α produces disruption of the blood–brain barrier. Brain Res..

[22] Welser-Alves J.V., Milner R. (2013). Microglia are the major source of TNF-α and TGF-β1 in postnatal glial cultures; regulation by cytokines, lipopolysaccharide, and vitronectin. Neurochem. Int..

[23] Ha J., Choi J., Kwon A., Kim K., Kim S.-J., Bae S.H., Son J.S., Kim S.-N., Kwak B.O., Lee R. (2018). Interleukin-4 and tumor necrosis factor-alpha levels in children with febrile seizures. Seizure.

[24] Zhao W., Xie W., Xiao Q., Beers D.R., Appel S.H. (2006). Protective effects of an anti-inflammatory cytokine, interleukin-4, on motoneuron toxicity induced by activated microglia. J. Neurochem..

[25] Hart Y.M., Andermann F., Robitaille Y., Laxer K.D., Rasmussen T., Davis R. (1998). Double pathology in Rasmussen’s syndrome: A window on the etiology?. Neurology.

[26] Lagarde S., Villeneuve N., Trébuchon A., Kaphan E., Lepine A., McGonigal A., Roubertie A., Barthez M.-A.J., Trommsdorff V., Lefranc J. (2016). Anti–tumor necrosis factor alpha therapy (adalimumab) in Rasmussen’s encephalitis: An open pilot study. Epilepsia.

[27] Varelas P.N., Mirski M.A. (2001). Seizures in the Adult Intensive Care Unit. J. Neurosurg. Anesthesiol..

[28] Castilla-Guerra L., Fernández-Moreno M.d.C., López-Chozas J.M., Fernández-Bolaños R. (2006). Electrolytes Disturbances and Seizures. Epilepsia.

[29] Hitchings A.W. (2016). Drugs that lower the seizure threshold. Adverse Drug React. Bull..

[30] Buchanan N. (2001). Medications which may lower seizure threshold. Aust. Prescr..

[31] Tesoro E.P., Brophy G.M. (2010). Pharmacological Management of Seizures and Status Epilepticus in Critically Ill Patients. J. Pharm. Pract..

[32] Hirsch L.J. (2004). Continuous EEG monitoring in the intensive care unit: An overview. J. Clin. Neurophysiol. Off. Publ. Am. Electroencephalogr. Soc..

[33] Drislane F.W., Lopez M.R., Blum A.S., Schomer D.L. (2008). Detection and Treatment of Refractory Status Epilepticus in the Intensive Care Unit. J. Clin. Neurophysiol..

[34] Kubota Y., Nakamoto H., Egawa S., Kawamata T. (2018). Continuous EEG monitoring in ICU. J. Intensive Care.

[35] Boly M., Maganti R. (2014). Monitoring epilepsy in the intensive care unit: Current state of facts and potential interest of high density EEG. Brain Inj..

[36] Vespa P.M., Nenov V., Nuwer M.R. (1999). Continuous EEG monitoring in the intensive care unit: Early findings and clinical efficacy. J. Clin. Neurophysiol. Off. Publ. Am. Electroencephalogr. Soc..

[37] Pandian J.D., Cascino G.D., So E.L., Manno E., Fulgham J.R. (2004). Digital video-electroencephalographic monitoring in the neurological-neurosurgical intensive care unit: Clinical features and outcome. Arch. Neurol..

[38] Drake M.E., Padamadan H., Newell S.A. (1998). Interictal quantitative EEG in epilepsy. Seizure.

[39] van Putten M.J.A.M., Tavy D.L.J. (2004). Continuous quantitative EEG monitoring in hemispheric stroke patients using the brain symmetry index. Stroke.

[40] Coates S., Clarke A., Davison G., Patterson V. (2012). Tele-EEG in the UK: A report of over 1,000 patients. J. Telemed. Telecare.

[41] Hobbs K., Krishnamohan P., Legault C., Goodman S., Parvizi J., Gururangan K., Mlynash M. (2018). Rapid Bedside Evaluation of Seizures in the ICU by Listening to the Sound of Brainwaves: A Prospective Observational Clinical Trial of Ceribell’s Brain Stethoscope Function. Neurocrit. Care.

[42] Waziri A., Claassen J., Stuart R.M., Arif H., Schmidt J.M., Mayer S.A., Badjatia N., Kull L.L., Connolly E.S., Emerson R.G. (2009). Intracortical electroencephalography in acute brain injury. Ann. Neurol..

[43] Yamazaki M., Terrill M., Fujimoto A., Yamamoto T., Tucker D.M. (2012). Integrating dense array EEG in the presurgical evaluation of temporal lobe epilepsy. ISRN Neurol..

[44] Yamazaki M., Tucker D.M., Fujimoto A., Yamazoe T., Okanishi T., Yokota T., Enoki H., Yamamoto T. (2012). Comparison of dense array EEG with simultaneous intracranial EEG for interictal spike detection and localization. Epilepsy Res..

[45] Hutchinson P.J., Hutchinson D.B., Barr R.H., Burgess F., Kirkpatrick P.J., Pickard J.D. (2000). A new cranial access device for cerebral monitoring. Br. J. Neurosurg..

[46] Tavakoli S., Peitz G., Ares W., Hafeez S., Grandhi R. (2017). Complications of invasive intracranial pressure monitoring devices in neurocritical care. Neurosurg. Focus.

[47] Ritter A.C., Wagner A.K., Fabio A., Pugh M.J., Walker W.C., Szaflarski J.P., Zafonte R.D., Brown A.W., Hammond F.M., Bushnik T. (2016). Incidence and risk factors of posttraumatic seizures following traumatic brain injury: A Traumatic Brain Injury Model Systems Study. Epilepsia.

[48] Kodankandath T.V., Farooq S., Wazni W., Cox J.-A., Southwood C., Rozansky G., Johnson V., Lynch J.R. (2017). Seizure Prophylaxis in the Immediate Post-Hemorrhagic Period in Patients with Aneurysmal Subarachnoid Hemorrhage. J. Vasc. Interv. Neurol..

[49] Yerram S., Katyal N., Premkumar K., Nattanmai P., Newey C.R. (2018). Seizure prophylaxis in the neuroscience intensive care unit. J. Intensive Care.

[50] Connolly E.S., Rabinstein A.A., Carhuapoma J.R., Derdeyn C.P., Dion J., Higashida R.T., Hoh B.L., Kirkness C.J., Naidech A.M., Ogilvy C.S. (2012). Guidelines for the Management of Aneurysmal Subarachnoid Hemorrhage: A Guideline for Healthcare Professionals from the American Heart Association/American Stroke Association. Stroke.

[51] Carney N., Totten A.M., O’Reilly C., Ullman J.S., Hawryluk G.W.J., Bell M.J., Bratton S.L., Chesnut R., Harris O.A., Kissoon N. (2017). Guidelines for the Management of Severe Traumatic Brain Injury. Neurosurgery.

[52] Temkin N.R., Dikmen S.S., Anderson G.D., Wilensky A.J., Holmes M.D., Cohen W., Newell D.W., Nelson P., Awan A., Winn H.R. (1999). Valproate therapy for prevention of posttraumatic seizures: A randomized trial. J. Neurosurg..

[53] Temkin N.R., Dikmen S.S., Wilensky A.J., Keihm J., Chabal S., Winn H.R. (1990). A randomized, double-blind study of phenytoin for the prevention of post-traumatic seizures. N. Engl. J. Med..

[54] Temkin N.R. (2001). Antiepileptogenesis and seizure prevention trials with antiepileptic drugs: Meta-analysis of controlled trials. Epilepsia.

[55] Diringer M.N., Bleck T.P., Claude Hemphill J., Menon D., Shutter L., Vespa P., Bruder N., Connolly E.S., Citerio G., Gress D. (2011). Critical Care Management of Patients Following Aneurysmal Subarachnoid Hemorrhage: Recommendations from the Neurocritical Care Society’s Multidisciplinary Consensus Conference. Neurocrit. Care.

[56] Alldredge B.K., Gelb A.M., Isaacs S.M., Corry M.D., Allen F., Ulrich S., Gottwald M.D., O’Neil N., Neuhaus J.M., Segal M.R. (2001). A comparison of lorazepam, diazepam, and placebo for the treatment of out-of-hospital status epilepticus. N. Engl. J. Med..

[57] Silbergleit R., Durkalski V., Lowenstein D., Conwit R., Pancioli A., Palesch Y., Barsan W., NETT Investigators (2012). Intramuscular versus intravenous therapy for prehospital status epilepticus. N. Engl. J. Med..

[58] Scheepers M., Scheepers B., Clarke M., Comish S., Ibitoye M. (2000). Is intranasal midazolam an effective rescue medication in adolescents and adults with severe epilepsy?. Seizure.

[59] Bhattacharyya M., Kalra V., Gulati S. (2006). Intranasal midazolam vs rectal diazepam in acute childhood seizures. Pediatr. Neurol..

[60] Buck M.L., Wiggins B.S., Sesler J.M. (2007). Intraosseous Drug Administration in Children and Adults During Cardiopulmonary Resuscitation. Ann. Pharmacother..

[61] Goodkin H.P., Sun C., Yeh J.-L., Mangan P.S., Kapur J. (2007). GABAA Receptor Internalization during Seizures. Epilepsia.

[62] Glauser T., Shinnar S., Gloss D., Alldredge B., Arya R., Bainbridge J., Bare M., Bleck T., Dodson W.E., Garrity L. (2016). Evidence-Based Guideline: Treatment of Convulsive Status Epilepticus in Children and Adults: Report of the Guideline Committee of the American Epilepsy Society. Epilepsy Curr..

[63] Kalss G., Rohracher A., Leitinger M., Pilz G., Novak H.F., Neuray C., Kreidenhuber R., Höfler J., Kuchukhidze G., Trinka E. (2018). Intravenous brivaracetam in status epilepticus: A retrospective single-center study. Epilepsia.

[64] Santamarina E., Carbonell B.P., Sala J., Gutiérrez-Viedma Á., Miró J., Asensio M., Abraira L., Falip M., Ojeda J., López-González F.J. (2019). Use of intravenous brivaracetam in status epilepticus: A multicenter registry. Epilepsia.

[65] Strzelczyk A., Steinig I., Willems L.M., Reif P.S., Senft C., Voss M., Gaida B., von Podewils F., Rosenow F. (2017). Treatment of refractory and super-refractory status epilepticus with brivaracetam: A cohort study from two German university hospitals. Epilepsy Behav. EB.

[66] Farrokh S., Bon J., Erdman M., Tesoro E. (2019). Use of Newer Anticonvulsants for the Treatment of Status Epilepticus. Pharmacother. J. Hum. Pharmacol. Drug Ther..

[67] Strzelczyk A., Zöllner J.P., Willems L.M., Jost J., Paule E., Schubert-Bast S., Rosenow F., Bauer S. (2017). Lacosamide in status epilepticus: Systematic review of current evidence. Epilepsia.

[68] Treiman D.M., Meyers P.D., Walton N.Y., Collins J.F., Colling C., Rowan A.J., Handforth A., Faught E., Calabrese V.P., Uthman B.M. (1998). A comparison of four treatments for generalized convulsive status epilepticus. Veterans Affairs Status Epilepticus Cooperative Study Group. N. Engl. J. Med..

[69] Farrokh S., Tahsili-Fahadan P., Ritzl E.K., Lewin J.J., Mirski M.A. (2018). Antiepileptic drugs in critically ill patients. Crit. Care.

[70] Lexicomp. https://online.lexi.com/lco/action/home.

[71] Garnett W.R. (2000). Clinical Pharmacology of Topiramate: A Review. Epilepsia.

[72] Wu Y.W., Shek D.W., Garcia P.A., Zhao S., Johnston S.C. (2002). Incidence and mortality of generalized convulsive status epilepticus in California. Neurology.

[73] Guidelines for Standard Order Sets. https://www.ismp.org/guidelines/standard-order-sets.

[74] Mahmoud S.H. (2017). Antiepileptic Drug Removal by Continuous Renal Replacement Therapy: A Review of the Literature. Clin. Drug Investig..

[75] Schetz M., Ferdinande P., Van den Berghe G., Verwaest C., Lauwers P. (1995). Pharmacokinetics of continuous renal replacement therapy. Intensive Care Med..

[76] Lewis J.H., Stine J.G. (2013). Review article: Prescribing medications in patients with cirrhosis—A practical guide. Aliment. Pharmacol. Ther..

[77] Cheng V., Abdul-Aziz M.-H., Roberts J.A., Shekar K. (2018). Optimising drug dosing in patients receiving extracorporeal membrane oxygenation. J. Thorac. Dis..

[78] Shekar K., Roberts J.A., Mcdonald C.I., Ghassabian S., Anstey C., Wallis S.C., Mullany D.V., Fung Y.L., Fraser J.F. (2015). Protein-bound drugs are prone to sequestration in the extracorporeal membrane oxygenation circuit: Results from an ex vivo study. Crit. Care Lond. Engl..

[79] Lynch T., Price A.L. (2007). The Effect of Cytochrome P450 Metabolism on Drug Response, Interactions, and Adverse Effects. Am. Fam. Physician.

[80] McElnay J.C., D’Arcy P.F. (1983). Protein binding displacement interactions and their clinical importance. Drugs.

[81] Schleibinger M., Steinbach C.L., Töpper C., Kratzer A., Liebchen U., Kees F., Salzberger B., Kees M.G. (2015). Protein binding characteristics and pharmacokinetics of ceftriaxone in intensive care unit patients. Br. J. Clin. Pharmacol..

[82] Lindow J., Wijdicks E.F. (1994). Phenytoin toxicity associated with hypoalbuminemia in critically ill patients. Chest.

[83] Wen Z.-P., Fan S.-S., Du C., Yin T., Zhou B.-T., Peng Z.-F., Xie Y.-Y., Zhang W., Chen Y., Xiao J. (2017). Drug-drug interaction between valproic acid and meropenem: A retrospective analysis of electronic medical records from neurosurgery inpatients. J. Clin. Pharm. Ther..

[84] Suzuki E., Yamamura N., Ogura Y., Nakai D., Kubota K., Kobayashi N., Miura S., Okazaki O. (2010). Identification of Valproic Acid Glucuronide Hydrolase as a Key Enzyme for the Interaction of Valproic Acid with Carbapenem Antibiotics. Drug Metab. Dispos..

[85] Bede P., Lawlor D., Solanki D., Delanty N. (2016). Carbapenems and valproate: A consumptive relationship. Epilepsia Open.

[86] Klotz U. (2009). Pharmacokinetics and drug metabolism in the elderly. Drug Metab. Rev..

[87] Hutchison L.C., O’Brien C.E. (2007). Changes in Pharmacokinetics and Pharmacodynamics in the Elderly Patient. J. Pharm. Pract..

[88] Batchelor H.K., Marriott J.F. (2015). Paediatric pharmacokinetics: Key considerations. Br. J. Clin. Pharmacol..

[89] Loebstein R., Lalkin A., Koren G. (1997). Pharmacokinetic Changes During Pregnancy and Their Clinical Relevance. Clin. Pharmacokinet..

